# Anti-Typhoid Inoculation in the Army

**Published:** 1914-09-26

**Authors:** 


### ANTI-TYPHOID INOCULATION IN THE ARMY.

We welcome very heartily the communications which have appeared in the press relative to the subject of inoculation against typhoid fever. These are the very agencies which are needed for the education and establishment of a strong public opinion in the community, and it is mainly on such an opinion that, in the present juncture, the practice must rest for support. Voices are heard which claim the power of the State and the force of compulsion. But the present is no time for such advocacy. What the country wants at the moment is trained and armed men, and this is not the occasion to insist that every one of these shall submit to a procedure which to many of them will appear mysterious, and therefore more or less suspect. Whether compulsion in such a matter is advisable at any time is a question open to argument. But in the circumstances in which we stand the answer to the question here and now must be decidedly in the negative. The stress is upon us for a growing Army, and anything which hinders this is a danger and a snare. It is neither possible nor advisable to demand that every one of the new recruits shall submit to anti-typhoid inoculation.

Leaving, then, the question of compulsion on one side, and recognising the strong evidence there is in favour of the practice of anti-typhoid inoculation, it is well to consider by what means the education of those immediately concerned may be effected. In the first place we recognise, as already stated, the value of authorised statements in the press. These engage, in greater or less measure, the thoughts of readers who in many instances are anxious concerning the welfare of relatives now serving in His Majesty's forces. Naturally it is to the more thoughtful and more educated part of the community to which such statements appeal, and their force is therefore carried particularly to the officers rather than to the rank and file. But to gain the officers is to gain much. Let these be in a position to say, and to show by example, that they believe in the protective value of anti-typhoid inoculation, and there will be abundant followers among their men. It is the taught who profit by teaching, and to secure a public opinion in the Army it is the leaders who must first be secured.

There is a further educational influence which may be evoked. The Expeditionary Force has business in hand even more important than anti-typhoid inoculation. But at home among the Territorials and the new recruits medical officers must have many chances of exercising influence and securing assent. We should like to see every one of these officers provided with a brief statement of the scientific and statistical evidence in favour of the practice here considered. Military demands must, of course, take the first place, but surely among the troops at home many might be persuaded of the personal advantages which inoculation would secure for them. A couple of days' absence from drill can hardly be a serious matter if the men are taken in small groups, so that at no one time is the strength of the unit substantially weakened.

Unless all history is misleading, where there are armies there will certainly sooner or later be typhoid, and a pitiful list of victims will follow. Sanitary precautions are of the highest importance, and could they be universally enforced all might be well. But under the stress of military necessity, or by the carelessness of individuals, such precautions invariably break down. It is morally certain that this experience will recur. When it does recur, inoculation may save the individual from disaster. Therefore every soldier should at least have the choice of such protection as inoculation affords.

